# Constructing Photoactive Au NP/MXene–BiOCl Moiré Superlattice Nanosheets for Photoelectrochemical Detection of Protein Kinase Activity

**DOI:** 10.3390/ijms26031348

**Published:** 2025-02-05

**Authors:** Yansen Li, Jingyao Chen, Chaojie Yang, Wenhao Fan, Qirong Chen, Nan Yang, Pingye Deng, Wenlei Zhai, Zhiyong Yan, Feng Wang

**Affiliations:** 1 Department of General Surgery, Beijing Chaoyang Hospital, Capital Medical University, Beijing 100020, China; 2 Institute of Analysis and Testing, Beijing Center for Physical and Chemical Analysis, Beijing Academy of Science and Technology, Beijing 100089, China; 3 Institute of Quality Standard and Testing Technology, Beijing Academy of Agriculture and Forestry Sciences, Beijing 100097, China; 4 Shenzhen Institute of Advanced Technology, Chinese Academy of Sciences, Shenzhen 518055, China

**Keywords:** photoelectrochemical sensor, protein kinase activity, MXenes, Moiré superlattice nanosheets, point-of-care testing

## Abstract

A novel photoelectrochemical (PEC) biosensor was proposed by preparing Au NP/MXene–BiOCl Moiré superlattice nanosheets as the probes. Upon irradiation with visible light, the probe exhibited excellent electrical conductivity as well as high photoelectric conversion efficiency. Benefitting from the excellent PEC property of the hybrid probe, sensitive and accurate detection of protein kinase activity was demonstrated with a limit of detection of 0.0029 U mL^−1^. This study verifies the great PEC potential of MXene hybrid nanomaterials.

## 1. Introduction

Protein kinases play a vital role in cellular signal transduction [1]. These enzymes regulate cell proliferation, differentiation, apoptosis, metabolism and gene transcription by catalyzing protein phosphorylation [2,3]. Pathological and pharmacological studies have verified that abnormal protein kinase activity (PKA) is the cause of various diseases, including tumor growth, inflammation, diabetes, cardiovascular diseases, and disorders of the nervous system [4,5]. Thus, PKA can serve as an important biomarker for the early diagnosis of many diseases, including certain types of cancer [6,7]. As a result, the accurate monitoring of protein kinase activity has become a hot topic in the field of analytical chemistry and clinical diagnosis.

At present, a variety of sensing methods have been developed for the detection of protein kinase activity, including colorimetric detection [8], fluorescence sensors [9], mass spectrometry [10], electrochemical sensors [11] and photoelectrochemical (PEC) sensors [12]. Among them, the PEC sensing strategy is based on using the photovoltaic characteristics of semiconductors to convert optical signals into electrical signals [13,14]. By modifying specific recognition elements on the PEC interface, biosensors can be constructed for the sensitive and real-time monitoring of target molecules. Due to the separative forms of excitation (light) and signal output (current), the background noise of the PEC sensor is extremely low [15]. Meanwhile, it also presents other advantages, including fast response, ultrahigh sensitivity, low cost and being a portable device. Benefitting from these merits, PEC sensors have been widely used in biomedical analysis, environmental monitoring, food safety inspection and so forth [16,17,18].

For the monitoring of protein kinase activity using PEC sensors, previous studies have been conducted. For example, a PEC sensor has been reported with the use of g-C_3_N_4_ and TiO_2_ hybrid nanomaterials as light-responsive substrates. The sensitive detection of protein kinase activity can be realized through dendritic PAMAM-induced signal amplification [19]. Our previous study also employed the localized surface plasmonic resonance (LSPR) effect of Au nanoparticles (Au NPs) and the dye-sensitization effect to enhance the signal [20]. However, some shortcomings of current PEC sensors need to be overcome to guarantee ultrahigh sensitivity and reliability for the assay of complicated clinical samples. Firstly, the photoelectric conversion efficiency of photoactive nanoprobes needs to be improved. At present, commonly used probes include quantum dots, organic dyes, metal nanoparticles and so forth [21,22,23,24,25]. However, the yields of photogenerated carriers made from these nanomaterials were relatively low. In addition, the recombination of photogenerated electrons and holes led to low photoelectric conversion efficiency. Thirdly, the active sites on the surface of the photoactive probes were limited, with a small proportion of the active sites participating in the photocatalytic reaction. These reasons compromised the sensitivity and stability of PEC sensors, thus hindering their application in clinical sample analysis. As a result, there is an urgent need to investigate novel photoactive probes for the construction of a PEC sensing interface. Ideally, the new PEC sensing interface should exhibit both improved photoelectric conversion efficiency and highly specific recognition of phosphorylation catalyzed by protein kinases.

MXenes are a class of metallic carbon/nitrides with two-dimensional layered nanostructures. Since an earlier report in 2011, these group of nanomaterials have been extensively studied and used in many areas, including the development of new batteries and electrochemical sensors [26,27,28]. MXenes present the metallic conductivity of transition-metal carbides due to the presence of hydroxyl groups or terminal oxygen on their surfaces [29]. For biosensing, the advantages of MXenes include their large surface area, biocompatibility and so forth. Among MXenes, Ti_3_C_2_ MXenes are the most used, and their surfaces contain an intact metal atomic layer—Ti^2+^. Therefore, Ti_3_C_2_ MXenes exhibit excellent electrochemical performance as an electrode material for biosensors [30,31] and are suitable for application in PEC sensors as well [32,33]. Furthermore, Moiré superlattice bismuth oxychloride (BiOCl) spiral nanosheets, as a new promising ternary compound semiconductor, have also been utilized in our research due to their unique and excellent photocatalytic properties. Common BiOCl possesses unique layered crystal structures, which are composed of interlacing [Bi_2_O_2_] slabs with double halogen slabs. This structure contributes to the increasing separation efficiency of photoinduced electrons and holes. However, the light absorption capability of traditional BiOCl is not satisfactory owing to its wide band gap, which makes it difficult to activate under visible-light irradiation [34]. To overcome this issue, Moiré superlattice BiOCl spiral nanosheets are introduced in the current research. Due to the emergence of the Moiré superlattice, the nanosheet significantly reduces the band gap, which expands the light-absorbing capability of BiOCl from the ultraviolet range to the visible range [35]. Additionally, it effectively inhibits the recombination of photoexcited electrons and holes in the BiOCl structure, thereby increasing the photoelectric separation efficiency.

**Scheme 1:**
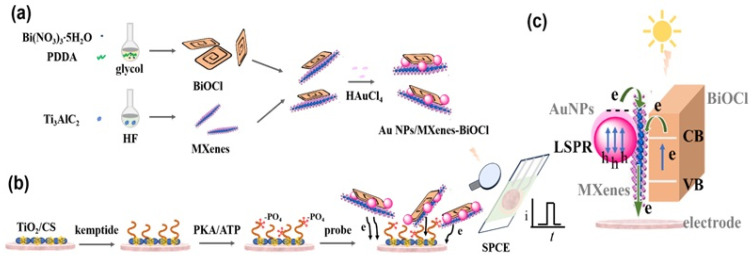
Schematic illustration of (**a**) the synthesis of Au NP/MXene–BiOCl Moiré superlattice nanosheet probes; (**b**) the fabrication process of the PEC biosensor; (**c**) the detailed separation and transfer mechanisms of photoinduced electrons from the probe when irradiated by visible light.

Herein, we introduce a PEC biosensor for the highly sensitive analysis of PKA, with outstanding biocompatibility and chemical stability. In the presence of protein kinase, the kemptide modified on the chitosan (CS)/TiO_2_-modified electrode can be phosphorylated and subsequently combined with the Au NPs/MXene–BiOCl by virtue of the interaction between the Ti ions and phosphate groups [36]. Under the irradiation of visible light, photocurrent was simultaneously generated, and the signal intensity was associated with the PKA. As a result, a quantitative analysis of kinase activity can be achieved. This method could be applied for the detection of PKA in various complex cell lysates, along with inhibitor screenings. The principle behind the proposed label-free PEC biosensor for PKA is illustrated in Scheme 1. Firstly, the kemptide was anchored to the CS/TiO_2_-modified screen-printing carbon electrode (SPCE). Subsequently, the electrode was treated with MCH to seal the unoccupied sites on the electrode’s surface. After being catalyzed by PKA, the phosphorylated kemptide was chelated to Au NP/MXene–BiOCl probes by a metal–chelate interaction between the Ti ions on the basal plane of the MXenes and the phosphate groups from the phosphorylated kemptide. When exposed to visible light, excited electrons were generated via a localized surface plasma resonance effect on the Au NPs, as well as the conduction band of spiral BiOCl Moiré superlattice nanosheets. Then, these excited electrons were transferred to the MXenes and generated the photocurrent for the PEC assay.

## 2. Results and Discussion

### 2.1. Design of the PEC Biosensor

In the current system, the superior photoelectric conversion efficiency of Au NP/MXene–BiOCl nanocomposites is attributed to the following points: (1) Ti_3_C_2_ Tx MXenes acted as specific photoactive phosphate group recognition elements, as well as reducing agents; thus, a large number of Au NPs were synthesized on MXene–BiOCl nanocomposites, and then all the Au NP/MXene–BiOCl probes were efficiently captured by the phosphorylated kemptide. (2) MXene is an ideal support owing to its uniform size and large surface area, thus avoiding the aggregation of BiOCl and AuNPs, which can expose more active sites. Upon irradiation with light, the electrons of the Au NP/MXenes–BiOCl composite can be excited in two ways: One is the photogenerated electrons’ transition from the valance band (VB) of BiOCl to the conduction band (CB) of BiOCl. The electrons in the CB are further transferred to MXenes in the Au NP/MXene–BiOCl composite. The other way is that electrons are generated by the LSPR of Au NPs, and these electrons are transferred to the MXenes immediately, which results in the enhancement of the photocurrent. Ti_3_C_2_ can work as an electron-trapping and shuttling site, which helps to suppress the recombination of electrons/holes and promotes the separation and transfer of electrons. The strong interaction between the semiconducting BiOCl and Ti_3_C_2_ Tx MXenes and the excellent electrical conductivity of AuNPs facilitated the transfer of electrons and resulted in preventing the recombination of photogenerated electron–hole pairs. As a result, the photoelectric conversion efficiency can be remarkably improved.

### 2.2. Characterization of the Synthesized Au NP/MXene–BiOCl Nanocomposites

**Figure 1:**
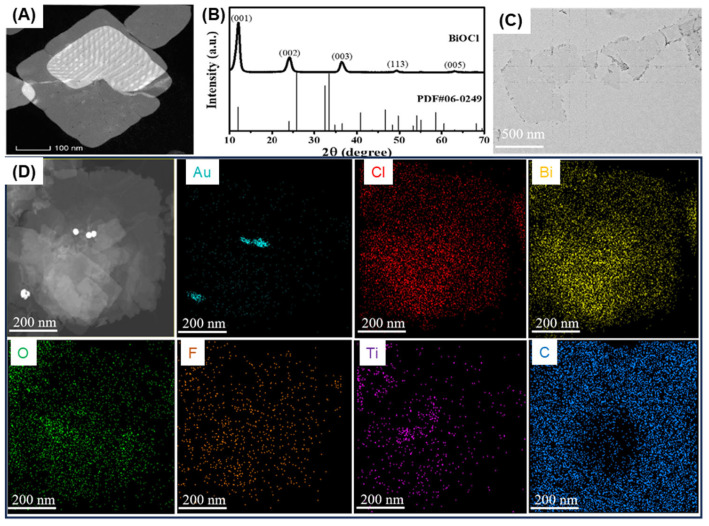
The HRTEM images. (**A**) Powder XRD patterns (**B**) of the molar superlattice BiOCl spiral nanosheets. TEM images (**C**) of the MXenes and TEM (**D**) of the Au NP/MXene–BiOCl nanocomposites.

The Au NP/MXene–BiOCl nanocomposites significantly contribute to the amplification of PEC signals and the recognition of phosphate groups on the sensing platform. The morphological characteristics of the synthesized two-dimensional Moiré superlattice BiOCl spiral nanosheets, MXenes and Au NP/MXene–BiOCl samples were confirmed. As shown in Figure 1A and Supplementary Figure S1, the existence of a Moiré pattern in High-Resolution Transmission Electron Microscopy (HRTEM), Scanning Transmission Electron Microscopy (STEM) and crystal diffraction and the existence of the angle of interlayer diffraction spots prove the successful preparation of two-dimensional molar superlattice BiOCl spiral nanosheets. The XRD of the prepared two-dimensional molar superlattice BiOCl spiral nanosheets was consistent with the reported molar superlattice BiOCl spiral nanosheets, and only the (001) crystallographic plane was exposed (Figure 1B). The absence of certain peaks in the XRD spectra compared with the standard PDF card of BiOCl is attributed to the broken in-plane crystal periodicity with the emergence of the Moiré pattern in the incommensurately twisted stacking of the nanosheets [35]. Both SEM and AFM showed relatively uniform spiral nanosheets (Figure S1). Figure 1C shows the sheet structure of MXenes. Figure 1D shows the TEM images of the Au NP/MXene–BiOCl nanocomposites, respectively. It can be clearly observed that the Au NPs on the MXene–BiOCl resemble a regular shape with an average diameter of 80 nm. In addition, the corresponding EDX elemental mapping images (Figure 1D and Table S1) of Ti, Au, Bi, O, Cl, F, and C in the Au NPs/MXene–BiOCl were taken and the EDX analysis (Figure S3) was performed to investigate the elemental compositions of the Au NP/MXene–BiOCl probes. The XRD pattern of the Au NPs/MXenes–BiOCl revealed diffraction peaks, which corresponded to diffractions from the (111), (220), (200) and (311) planes of the Au NPs’ face-centered cubic structure, as detailed in Supplementary Figure S2. Moreover, the intensity of the (002) peak in the Au NPs/MXene–BiOCl was decreased due to the oxidation of MXene nanosheets, as they reacted with the Au precursor to generate Au NPs [37].

### 2.3. Electrochemical and PEC Responses of the Biosensor

**Figure 2:**
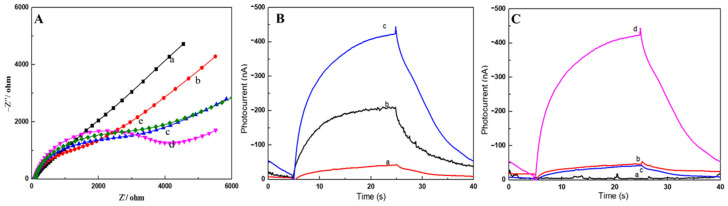
(**A**) EIS of (a) the bare GCE, (b) CS/TiO_2_/GCE, (c) kemptide/CS/TiO_2_/GCE, (d) phosphorylated kemptide/CS/TiO_2_/GCE, and (e) Au NP/MXene–BiOCl/phosphorylated kemptide/Au NPs/MXenes/GCE. The spectra data were captured in an [Fe (CN)_6_]^4−/3−^ solution over a frequency spectrum ranging from 0.1 Hz to 100 kHz. (**B**) The transient photocurrent responses of (a) phosphorylated kemptide with the treatment of MXenes (a), Au NPs/MXenes (b) and Au NP/MXene–BiOCl probes (c). (**C**) The transient photocurrent responses of (a) the bare SPCE, (b) kemptide/CS/TiO_2_/SPCE, (c) phosphorylated kemptide/CS/TiO_2_/SPCE, and (d) Au NP/MXene–BiOCl/phosphorylated kemptide/CS/TiO_2_/SPCE. The light power density is 190 mW cm^−2^.

Figure 2A shows the electrochemical impedance spectroscopy (EIS) of a glass carbon electrode (GCE) (curve a), a CS/TiO_2_-modified GCE (curve b), a kemptide/CS/TiO_2_-modified electrode (curve c), a phosphorylated kemptide/CS/TiO_2_-modified GCE (curve d) and a Au NP/MXene–BiOCl/phosphorylated kemptide/CS/TiO_2_-modified GCE (curve e) in [Fe (CN)_6_]^4−/3−^ solution in a frequency range from 0.1 Hz to 100 kHz. EIS stands out as an effective tool that provides information about impedance changes on an electrode’s surface caused by chemical modification. The diameter of the semicircle in the EIS plot is equal to the resistance to electron transfer (Rct), indicating the electron-transfer kinetics of the redox probe at the electrode–electrolyte interface. As shown in Figure 2A, the bare GCE exhibited a small Rct. Upon the deposition of CS/TiO_2_ onto the electrode, a noticeable enhancement of the photocurrent was detected (curve b). Following incubation in the kemptide solution, the EIS data of the resulting assembled layer displayed an elevated Rct value due to the kemptide’s coverage on the electrode surface, which hindered the access of redox probes to the electrode interface (curve c). The diameter of the semicircle remarkably increased after the phosphorylation of the kemptide by PKA, suggesting higher electron-transfer resistance at the electrode interface (curve d). When treated with the conductive Au NP/MXene–BiOCl probes, the Rct value for the resulting electrode decreased rapidly (curve e), signifying the effective assembling of the biosensor. Figure 2B presents the superiority of the proposed probes. After treating the phosphorylated kemptide/CS/TiO_2_-modified electrode with MXenes, a weak photocurrent signal could be observed, while the AuNPs/MXenes produced a higher photocurrent signal. However, when the AuNP/MXene–BiOCl probes were anchored to the modified electrode, the current signal was remarkably enhanced. These experiments verified the outstanding performance of the probes.

As presented in Figure 2C, for the bare screen-printed electrode (curve a) and kemptide/CS/TiO_2_/bare SPCE (curve b), there is a negligible PEC response. After being phosphorylated by PKA, similar results were observed (curve c). Upon modifying the phosphorylated kemptide-treated electrode with Au NP/MXene–BiOCl via the binding between Ti-O-P, a significant enhancement in PEC intensity could be observed (curve d), demonstrating the successful preparation of the biosensor.

### 2.4. Optimization and Establishment of the PEC Biosensor for the Detection of PKA

**Figure 3:**
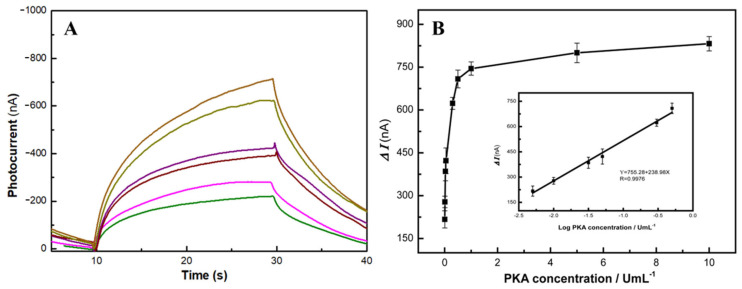
(**A**) The variation in photocurrent in a 0.1 M phosphate-buffered saline solution (PBS) supplemented with 0.1 M ascorbic acid and varying concentrations of PKA, ranging from 0.005 to 0.5 U mL^−1^, the corresponding concentration of each curve is 0.005 (green), 0.010 (pink), 0.030 (wine red), 0.050 (purple), 0.300 (dark yellow), 0.500 (brown). (**B**) The photocurrent responses at PKA concentrations spanning from 0 to 10 U mL^−1^. The inset illustrates the linear correlation between ΔI and PKA. The light power density was maintained at 190 mW cm^−2^.

The PKA assessment was carried out under optimized conditions (Figure S4). Figure 3A illustrates the PEC signals over time during potential scanning at varying concentrations of PKA. The intensity of the PEC signal increased correspondingly with the increase in PKA concentration. Figure 3B shows the PEC intensities and the signal-to-noise ratios at different concentrations of PKA. The photocurrent signal intensities increased with the rising activity of PKA, ranging from 0.005 U mL^−1^ to 10 U mL^−1^. As depicted in the inset of Figure 3B, the photocurrent intensity was directly proportional to the logarithm value of PKA concentrations ranging from 0.005 U mL^−1^ to 0.5 U mL^−1^, adhering to the correlation equation of ΔI = 238.98 × log cPKA + 755.28 (R^2^ = 0.9976), where ΔI represents the photocurrent intensity and cPKA denotes the kinase activity. The determined detection limit for PKA was calculated to be 0.0029 U mL^−1^ using the formula LOD = (3σ − b)/K, where σ denotes the standard deviation of a blank value, b is the intercept and K represent the slope of the linear regression equation, respectively. This value indicated comparable performance compared with previously reports [9,20,36,38,39,40,41] (Table S2), implying that the novel PEC biosensor is suitable for the precise analysis of PKA. To evaluated the reproducibility of the proposed PEC sensing method, the same concentration of PKA of 0.15 U mL^−1^ was measured using six distinct biosensors. The relative standard deviation was determined as 4.52%, indicating relatively good reproducibility. The stability of the biosensor was also evaluated by analyzing the PKA at 0.1 U mL^−1^ with a freshly prepared electrode, and the RSD was 3.51%, indicating the good stability of the biosensor. The stability of the Au NP/MXene–BiOCl Moiré superlattice nanosheets and the firmness of the chemical identification of phosphate groups make the sensor outstanding in terms of reproducibility and stability. Furthermore, to study the selectivity of the PEC biosensor, the effects of different enzymes and proteins (transferritin, BSA, thrombin, T4 PKN) on the sensitivity of the biosensor were tested (Figure S5), and the results showed the good selectivity of the proposed platform.

### 2.5. Application in Real Sample Analysis

**Figure 4:**
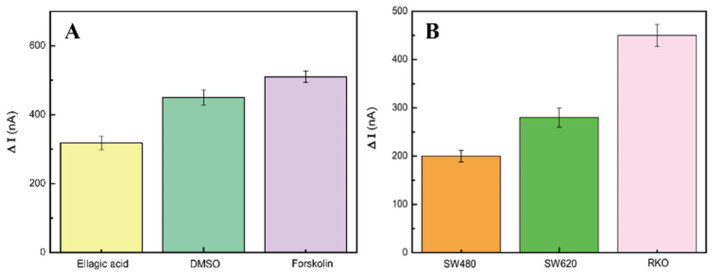
(**A**) The photocurrent measurements reflecting PKA expression in RKO cell lysates treated with ellagic acid, forskolin/IBMX and DMSO, respectively. (**B**) The photocurrent intensity corresponding to varying levels of PKA expression in SW620, SW480 and RKO cell lines. The error bars denote the standard deviation based on three parallel experiments.

PKA is pivotal in regulating the process of protein phosphorylation and has a significant impact on various cell activities. As presented in Figure 4A, the cell lysate treated with forskolin/IBMX generated the highest photocurrent signal, indicating enhanced PKA due to stimulation. In contrast, after treatment with the PKA inhibitor ellagic acid, the weakest photocurrent signal was observed. These results verified that the PEC biosensor provides a sensitive and effective approach in the quantitative analysis of PKA in cell lysates. As shown in Figure 4B, RKO cells demonstrated the most significant change in photocurrent, indicating the highest level of PKA among the tested cell lines. Conversely, SW480 cells produced the weakest change in photocurrent, suggesting the lowest level of PKA. This outcome was consistent with our previous experimental results and suggested that the proposed PEC biosensor is feasible for kinase analysis in cell samples.

## 3. Materials and Methods

### 3.1. Reagents and Materials

The screen-printed carbon electrode (SPCE) was provided by Poten Co., Ltd. (Beijing, China). Cysteine-terminated kemptide (CLRRASLG) was purchased from GL Biochem (Shanghai, China). Ellagic acid, forskolin, protein kinase A, adenosine 5′-triphosphate disodium salt (ATP) and 3-isobutyl-1-methylxanthine (IBMX) were obtained from Sigma-Aldrich (Burlington, VT, USA). Polydiallyl dimethyl ammonium chloride (PDDA, MW: 300,000–400,000), 6-hydroxy-1-hexanethiol (MCH), radioimmunoprecipitation assay (RIPA), dimethyl sulfoxide (DMSO) and other reagents were ordered from Aladdin Chemistry Co., Ltd. (Shanghai, China) with analytical-grade standard purity. Human colon cancer cells (SW480, SW620, and RKO) were sourced from the American Type Culture collection (ATCC) (Manassas, VA, USA). Fetal bovine serum (FBS) was supplied by Thermo Fisher Scientific Inc. (Waltham, MA, USA). The compound 3-isobutyl-1-methylxantine (IBMX) was procured from Sigma-Aldrich. Additional analytical-grade regents were furnished by Beijing Chemical Company (Beijing, China). All chemicals were utilized as received without any further purification.

### 3.2. Synthesis of Moiré Superlattice BiOCl Spiral Nanosheets and Ti_3_C_2_ MXene Nanosheets

The Moiré superlattice BiOCl spiral nanosheets were synthesized according to a previously reported method with slight modifications [35]. Briefly, 196 mg of bismuth nitrate and 10 mL of PDDA were added to 160 mL of ethylene glycol, heated by reflux at 200 °C for 2 h, cooled to room temperature, centrifuged and washed with water several times to obtain the BiOCl solid.

Preparation of Ti_3_C_2_ MXene nanosheets: Ti_3_AlC_2_ (1 g) powder was added to 15 mL of HF with a mass fraction of 48% and stirred at 45 °C for 24 h. Then, the solution was centrifugally washed, precipitated and dried at room temperature. After drying, layered Ti_3_C_2_ MXenes were obtained [37].

Preparation of Ti_3_C_2_ MXene–BiOCl nanocomposites: Five milliliters of synthesized Ti_3_C_2_ MXenes was sonicated with 11 μg/mL of BiOCl for 2 h. Then, the Ti_3_C_2_ MXenes–BiOCl was formed due to electrostatic adsorption.

### 3.3. Synthesis of Au NP/MXene–BiOCl Nanocomposite

Au NP/MXene–BiOCl nanocomposites were obtained by dropping 5 mL of MXene–BiOCl in 100 mL of 0.01% (*w*/*v*) HAuCl_4_ solution under gentle stirring for 1 min. After centrifugation and washing, the Au NP/MXene–BiOCl nanocomposite probes were synthesized and stored in sealed containers.

### 3.4. Apparatus and Characterization

EIS and photocurrent measurements were conducted using a CHI 660E electrochemical workstation (Shanghai Chenhua Apparatus Corporation, Shanghai, China). The experimental setup for these measurements comprised a standard three-electrode system, with the modified SPCE serving as the working electrode, a platinum wire as the counter electrode, and an Ag/AgCl electrode in a saturated KCl solution as the reference electrode. For photocurrent measurements, a PBS (0.1 M, pH 7.4) solution containing 0.1 M of ascorbic acid was utilized as the electrolyte. EIS measurement was performed in a three-electrode system using 0.1 M of KCl solution mixed with 5 mM of K_3_[Fe(CN)_6_]/K_4_[Fe(CN)_6_] (1:1 ratio) as an electrolyte, and the frequency range was set from 0.1 Hz to 100 kHz. An SXE300 Xe light source equipped with a 420 nm filter was utilized as the light source (Beijing Perfect light Technology Co., Ltd., Beijing, China). SEM imaging was performed on a Quanta 650 instrument (FEI, Hillsboro, OR, USA). X-ray diffraction (XRD) analysis of the synthesized materials was conducted using a D8 Advance (Bruker, Germany). TEM images and energy-dispersive X-ray (EDX) elemental mapping images were acquired with an F30S microscope (FEI, Hillsboro, OR, USA).

### 3.5. Biosensor Fabrication

Firstly, 20 μL of chitosan-modified TiO_2_ (CS/TiO_2_) solution was dropped onto a clean screen-printed electrode. Once dried, the CS/TiO_2_-coated electrode was kept in the dark and treated with a PBS solution (10 mM, pH 7.4) that included 500 μM of cysteine-conjugated kemptide for 12 h at 4 °C. This was succeeded by a blocking step using a 1 mM MCH solution at ambient temperature for one hour. Subsequently, the electrodes were washed with PBS, after which they were exposed to various concentrations of PKA and ATP in a buffer composed of 50 mM of Tris-HCl and 20 mM of MgCl_2_ (pH 7.4) at a temperature of 37 °C. Afterward, the phosphorylated peptide-modified electrode was dipped in a solution containing Au NP/MXene–BiOCl probes at room temperature. Finally, the modified electrode was washed to remove the nonspecific Au NP/MXene–BiOCl and used for photocurrent experiments.

### 3.6. Cell Culture and Preparation of Protein Lysate

Briefly, SW620, SW480 and RKO cell lines were cultivated in RPMI 1640 medium enriched with 10% fetal bovine serum (FBS) at 37 °C under a 5% CO_2_ environment. The culture media were then replaced with 4 mL of fresh RPMI 1640. Subsequently, RKO cells were separately treated with PKA-inhibiting ellagic acid (in DMSO), forskolin and IBMX (PKA activator, in DMSO), each at a final concentration of 25 µM, while an equivalent volume of DMSO served as the control group. All cells were washed three times with PBS, followed by lysis in 80 mL of a buffer mixture (RIPA and PMSF at a ratio of 99:1) and transferred into a 1.5 mL nuclease-free centrifuge tube. The tubes were placed on ice and agitated every 5–10 min for thorough lysis over a 30 min period. After lysis, the mixture was centrifuged at 12,000× *g* for 30 min at 4 °C. The cleared supernatant was then preserved at −80 °C for later PKA assays. The protein concentration in the lysate was quantified, and the lysate was diluted fivefold for the PKA measurement.

## 4. Conclusions

In this study, a sensitive PEC biosensor for the detection of PKA was prepared using AuNP/MXene–BiOCl Moiré superlattice nanosheets as the probes. Au NP/MXene–BiOCl provided high photo-to-current conversion efficiency, high electrical conductivity and a large-surface-area immobilization matrix that enhanced the number of loading kemptide molecules and expedited the spatial charge separation at the same time. MXenes were chelated to phosphorylated kemptide by forming Ti-O-P bonds between the Ti ions on the basal plane of Ti_3_C_2_ Tx MXenes and phosphate groups in phosphorylated kemptide. Then, excited electrons generated by the AuNPs and Moiré superlattice BiOCl spiral nanosheets under visible-light irradiation can be transferred to the electrode to generate the photocurrent signal. The proposed PEC sensor exhibited outstanding performance with an ultrahigh sensitivity of 0.0029 U mL^−1^ to PKA. Therefore, the current work provides a promising platform for drug screening and the diagnosis of kinase-related disease.

## Data Availability

Data are contained within the communication or Supplementary Materials.

## Supplementary Materials

The following supporting information can be downloaded at https://www.mdpi.com/article/10.3390/ijms26031348/s1.
