# Supplementary material for: Constructing Photoactive Au NP/MXene–BiOCl Moiré Superlattice Nanosheets for Photoelectrochemical Detection of Protein Kinase Activity
Source: Int J Mol Sci. 2025 Feb 5;26(3):1348. doi: 10.3390/ijms26031348 (PMC11818166; doi:10.3390/ijms26031348)
Supplement: Supplementary file 1 [file ijms-26-01348-s001.zip › ijms-3421349-supplementary.pdf]

Supplementary Information

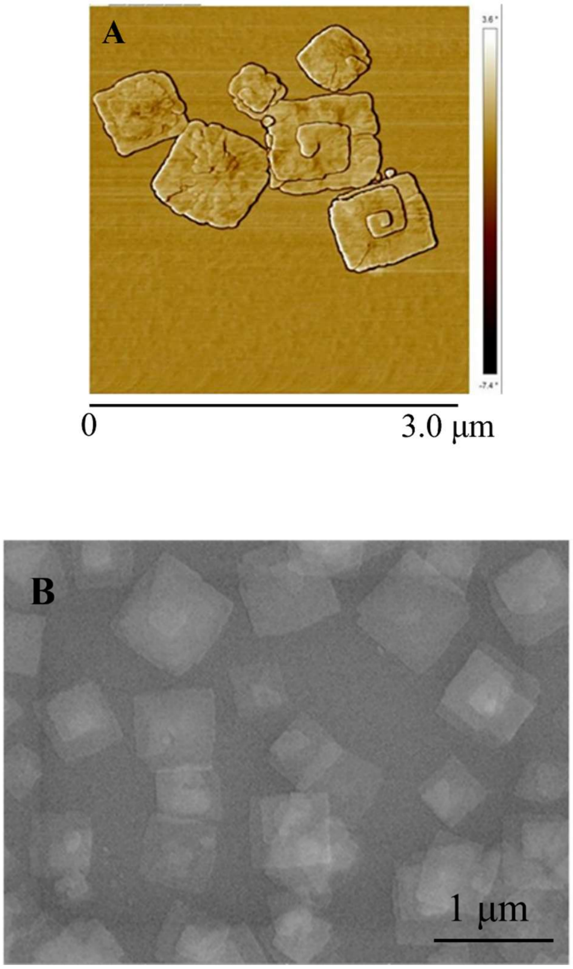

Supplementary Figure S1. The AFM image (A) and SEM image (B) of the Moiré superlattice BiOCl spiral nanosheets.

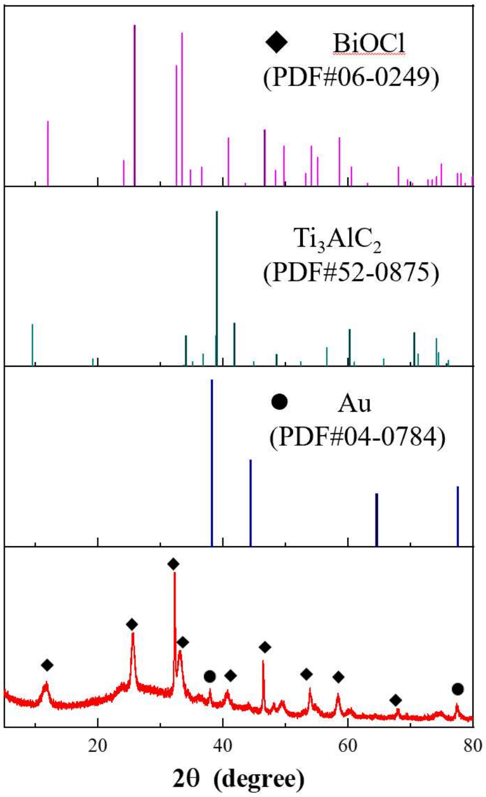

Supplementary Figure S2. Powder XRD patterns of simulated Au NPs/MXenes-BiOCl nanoparticles.

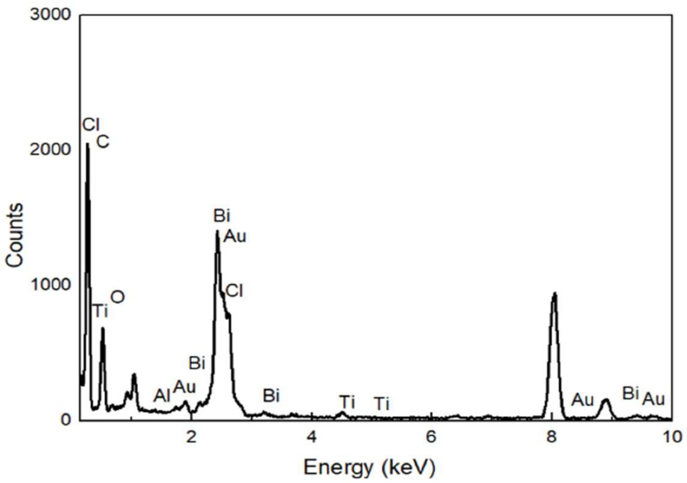

Supplementary Figure S3. the EDX analysis of Au NPs/MXenes-BiOCl nanoparticles.

Supplementary Table S1. Element weight percentage and atomic percent of Au NPs/MXenes-BiOCl nanoparticles.

Element	Weight percentage	Atomic percent
C K	37.66	75.75
O K	8.90	13.44
Al K	0.04	0.03
Cl K	7.71	5.25
Ti K	0.61	0.31
Au L	1.11	0.14
Bi M	43.97	5.08
Total	100.00	

Optimization of the detection conditions

The concentration of ATP is a key factor for PKA-catalyzed phosphorylation. As shown in Fig. S4A, the ECL intensity enhanced with higher concentration of ATP. The reaction reached to equilibrium with 120 μM ATP in the system. Therefore, 120 μM ATP was selected in the PKA activity measurement. The incubation temperature for Au NPs/MXenes-BiOCl on the modified electrode was another parameter for optimization. As shown in Fig. S5C, the optimal temperature for incubating Au NPs/MXenes-BiOCl probes was 37 ℃. As a result, 37 ℃ was determined as the incubation temperature in the current study. Additionally, the reaction time for phosphorylation also needs to be optimized. In Fig. S4C, with longer reaction time, the photocurrent signals increased accordingly and reached at the maximum level after 120 min, suggesting the phosphorylation reaction reached to its equilibrium. Thus, for phosphorylation, the reaction time was set as 120 min in subsequent experiments.

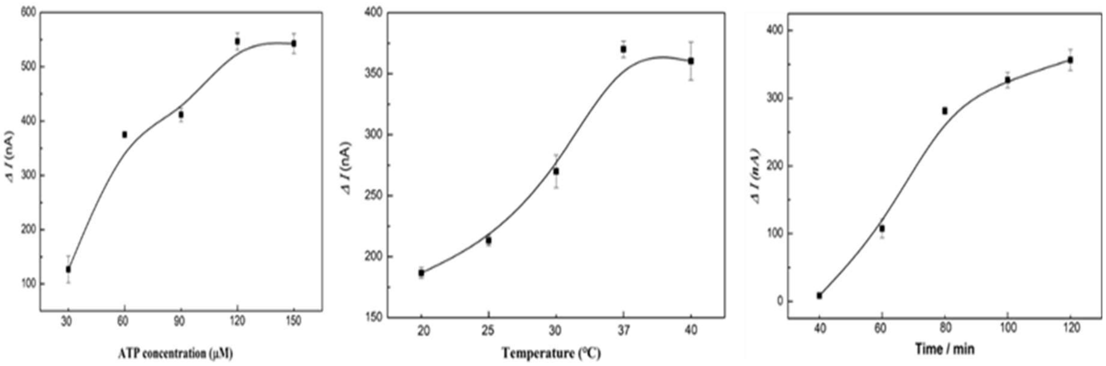

Supplementary Figure S4 Optimization of the experimental conditions. The influence of the ATP concentration **(A)** temperature for Au NPs/MXenes-BiOCl probes incubating on the phosphorylated kemptide **(B)**, and the incubation time **(C)**,

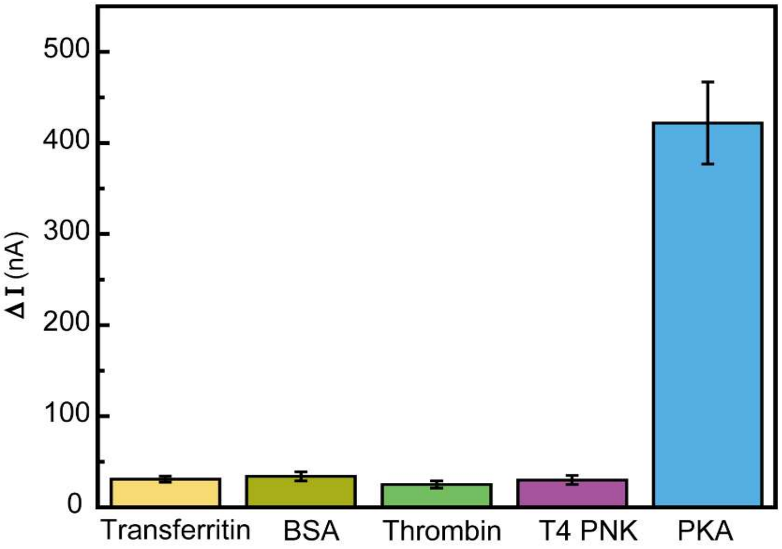

Supplementary Figure S5

Supplementary Table S2 Comparison of linear ranges and PKA detection limits by different detection methods.

Detection Methods	Linear range (UmL^{-1})	Detection limit (UmL^{-1})	Reference
Electrochemical	0.03-40	0.03	1
Electrochemical	0.005-500	0.002	2
Photoelectrochemical	0.008-1	0.005	3
Photoelectrochemical	0.005-0.0625	0.0049	4
Electrogenerated Chemiluminescence	0.015-40	0.001	5
Fluorescent	0.05-10	0.03	6

Photoelectrochemical	0.004-10	0.0026	7
Photoelectrochemical	0.005-10	0.0029	This work

Supporting references

1. Z. Wang, N. Sun, Y. He, Y. Liu and J. Li, *Anal. Chem.*, 2014, **86**, 6153-6159.
2. S. Congcong, L. Xiangzhi, R. Avraham, G. Linyan, Z. Kaina and Y. Minghui, *Biosens. Bioelectron.*, 2016, **85**, 220-225.
3. Z. Yan, Z. Wang, Z. Miao and Y. Liu, *Anal. Chem.*, 2015, **88**, 922-929.
4. W. Zonghua, Y. Zhiyong, W. Feng, C. Jibao, G. Lei, S. Jiakun and L. Yang, *Biosens. Bioelectron.*, 2017, **97**, 107-114.
5. Y. Sun, Y. Zhang, H. Zhang, M. Liu and Y. Liu, *Anal. Chem.*, 2020, **92**, 10668-10676.
6. Z. Liu, J. Bai, C. Liang, Y. Wang, H. Nie, X. Liu and H. Yan, *Biosens. Bioelectron.*, 2022, **203**, 114055.
7. Z. Yan, Y. Li, X. Wei, P. Li, J. Jiang, Y. Chen, P. Duan, X. Wang, P. Deng and X. Liu, *Biosens. Bioelectron.: X*, 2022, **11**, 100204.
